# Strategies for the Development of Bioprotective Cultures in Food Preservation

**DOI:** 10.1155/2022/6264170

**Published:** 2022-12-14

**Authors:** Luana Virgínia Souza, Evandro Martins, Isabella Maria Fernandes Botelho Moreira, Antônio Fernandes de Carvalho

**Affiliations:** Inovaleite—Department of Food Technology, Federal University of Viçosa (Universidade Federal de Viçosa) (UFV), Avenida Peter Henry Rolfs, s/n—Campus Universitário, Viçosa, MG 36570-900, Brazil

## Abstract

Consumers worldwide are increasingly demanding food with fewer ingredients, preferably without chemical additives. The trend called “Clean Label” has stimulated the development and commercialization of new types of bioprotective bacterial cultures. These bacteria are not considered new, and several cultures have been available on the market. Additionally, new bioprotective bacteria are being identified to service the clean label trend, extend the shelf life, and, mainly, improve the food safety of food. In this context, the lactic acid bacteria (LAB) have been extensively prospected as a bioprotective culture, as they have a long history in food production and their antimicrobial activity against spoilage and pathogenic microorganisms is well established. However, to make LAB cultures available in the market is not that easy, the strains should be characterized phenotypically and genotypically, and studies of safety and technological application are necessary to validate their bioprotection performance. Thus, this review presents information on the bioprotection mechanisms developed by LAB in foods and describes the main strategies used to identify and characterize bioprotective LAB with potential application in the food industry.

## 1. Introduction

According to the most recent reports, the “Clean Label Ingredients” market is projected to grow at a compound annual growth rate of 6.75% between 2021 and 2026, with the potential to reach up to USD 75.2 billion by 2026 [1]. During the COVID-19 pandemic, people have become more cautious with regard to healthy eating, especially for those who have been infected. The right choice of food can help balance the immune system and optimize its function. This explains the rapid growth of clean label foods in recent years [2].

The clean label trend has stimulated the food industry into developing new strategies for food production and preservation. Included in this scenario are the bioprotective bacterial cultures, which can answer the consumer's exigencies regarding foods with less ingredients [3–5].

The first definition of bioprotective bacterial cultures was proposed by Lücke in 1994 [6]. He defines them as being microbial cultures added to food for the unique purpose of inhibiting pathogens, extending shelf life, and improving their sensory quality. Later, the concept of biopreservation was introduced, and according to this new definition, bioprotection can be achieved through the addition of bioprotective cultures or their antimicrobial metabolites to promote extended shelf life and food safety [7, 8]. More recently, Vignolo and Fadda in 2015 unified the previous concepts [9]. Thus, biopreservation has come to be defined as the use of antagonistic microorganisms and/or their metabolites to inhibit undesirable microorganisms to increase the shelf life of food with minimal modifying its sensory properties.

To be considered a bioprotective culture, the bacterial strain needs to be identified at the genus and species level, have the GRAS (Generally Recognized as Safe) or QPS (Qualified Presumption of Safety) status, be stable and remain active under storage conditions, and inhibit the growth of pathogenic or/and spoilage microorganisms. In addition, for the commercialization of these cultures, proof of the intended technological effect is required, along with the definition of the quantity to be used and effectiveness at safe levels [10, 11].

Some works have highlighted that bioprotective bacteria are mainly classified as *Lactococcus*, *Lactobacillus*, *Lacticaseibacillus*, *Latilactobacillus*, *Lactiplantibacillus*, *Limosilactobacillus*, *Streptococcus*, *Leuconostoc*, *Weissella*, *Pediococcus*, *Carnobacterium*, and *Enterococcus*; with most of these belonging to the lactic acid bacteria group (LAB) (Table 1). LAB combined with non-LAB bacteria can be found in the market as bioprotective cultures for food application (Table 1).

The microorganisms of the LAB group have, as a common characteristic, the ability to produce lactic acid from carbohydrate fermentation, and they are commonly used in the production of fermented foods such as yogurts, cheeses, and fermented meats or vegetables [6, 8, 29, 40–42]. The LAB group is composed of the genera *Lactococcus*, *Streptococcus*, *Lactobacillus*, *Paralactobacillus*, *Holzapfelia*, *Amylolactobacillus*, *Bombilactobacillus*, *Companilactobacillus*, *Lapidilactobacillus*, *Agrilactobacillus*, *Schleiferilactobacil-lus*, *Loigolactobacilus*, *Lacticaseibacillus*, *Latilactobacillus*, *Dellaglioa*, *Liquorilactobacillus*, *Ligilactobacillus*, *Lactiplantibacillus*, *Furfurilactobacillus*, *Paucilactobacillus*, *Limosilactobacillus*, *Fructilactobacillus*, *Acetilactobacillus*, *Apilactobacillus*, *Levilactobacillus*, *Secundilactobacillus*, *Lentilactobacillus*, *Leuconostoc*, *Pediococcus*, *Aerococcus*, *Carnobacterium*, *Enterococcus*, *Oenococcus*, *Tetragenococcus*, *Vagococcus*, and *Weissella*; and some species such as *L. lactis*, *L. curvatus* and *L. plantarum* can be considered the GRAS status [8, 43]. The reported bioprotection effects of some LAB cultures may be affected by food factors such as pH, water activity, composition of the food matrix, processing type, storage conditions, and by microbial factors such as strain type, technological capacity of the strains, and genetic expression, resulting in significant changes in the efficiency of these bacteria in certain applications [4, 44, 45]. *L. mesenteroides* reduces the population of spoilage microorganisms from 4 to 5 log cycles when inoculated in apples, while a reduction of only 3 log cycles was observed when the same bacterium was added to lettuce [30]. In a study conducted by Mirkovic et al. [46], the LAB *L. lactis*, in addition to demonstrating an antimicrobial effect on *L. monocytogenes* and *S. aureus* in Quark-type cheese, also demonstrated an effect on filamentous fungi and yeasts from spontaneous growth during 21 days of product storage.

In an antifungal evaluation of the dairy systems model, Leyva Salas et al. [4] demonstrated that the antifungal activity of LAB was greater in cheese than in yogurt. These studies reinforce the idea that the bioprotection efficiency can change from one food to another, which reinforces the importance of the discovery of other bioprotective cultures that are able to cover all specificities of the food industry. By considering that LAB are a viable possibility to replace or reduce the number of preservatives in food and that few strains have been commercialized until now, research into alternative LAB cultures may help develop new processes and adapt technologies for the “clean label” demand. This review aims to describe the bioprotection mechanisms developed by LAB in foods and the main strategies used to identify LAB with potential application in the food industry.

## 2. Identified Bioprotection Mechanisms of LAB

**Figure 1:**
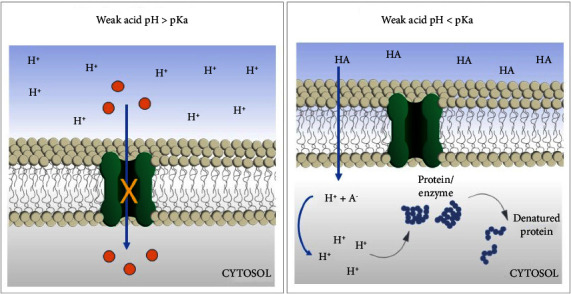
Mechanisms of weak acid.

The major preservative effects on food by LAB is associated with the rapid acidification of raw material due to the production and accumulation of mainly lactic acid. As it is a weak organic acid, with a pKa close to 3.0 and a pH greater than 3 in food, the antimicrobial activity of lactic acid is related to; the denaturation of membrane proteins, blocking transmembrane transport, proton gradient interference, enzyme inhibition, and reactive oxygen species (ROS) production, which disturbs the cell metabolism resulting in growth inhibition [36, 47–49]. However, higher antagonistic activity can be expected in food with high acidity (pH < 3.0), since the nondissociated form of acid prevails at these pH conditions. The nondissociated form of lactic acid is apolar and can cross through the cytoplasmatic membrane of the target microorganism, reaching the cytosol [50]. Once inside the cytoplasm, whose pH is close to neutral (pH > pKa), the lactic acid dissociates form and the release of hydrogen ions promotes the acidification of the cytoplasm. As a general consequence, the internal proteins are denatured and the enzymatic activities are interrupted, leading to the death of the microorganism (Figure 1) [51].

**Figure 2:**
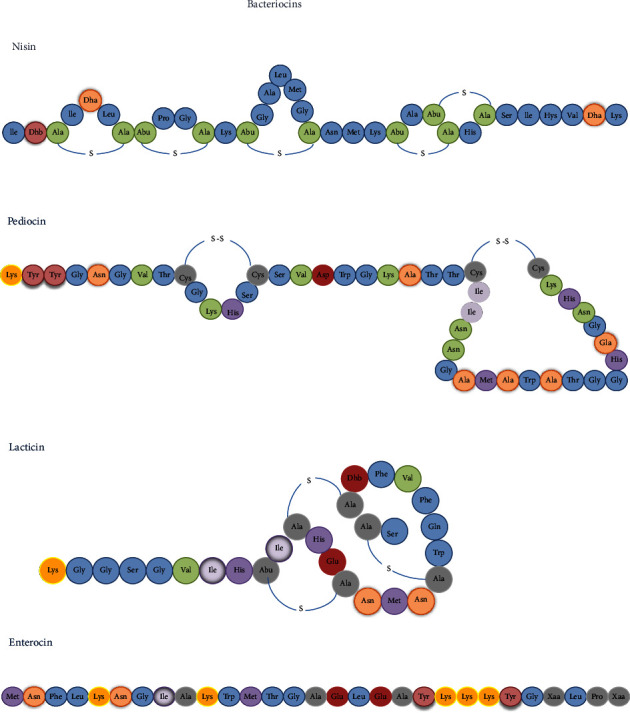
Bacteriocin molecules.

In addition to lactic acid production, the bioprotective cultures are responsible for producing various other compounds, including; other types of organic acids like acetic, benzoic, formic, succinic, phenyllactic, indole lactic, and azelaic acids, hydrogen peroxide, acetoin, diacetyl, reuterin, and peptides with antimicrobial activity such as bacteriocins (Figure 2). The action mechanisms of the organic acids produced by LAB are very close to the one described for lactic acid, because they are weak acids [50, 51]. However, some studies suggest that acetic acid, for example, can also inactivate other microorganisms by synergy or through another type of mechanism not yet elucidated [52].

Due to a low molecular weight and absence of charge, hydrogen peroxide crosses the membrane of the target microorganism and reaches the cytoplasm where it is reduced and decomposed to the hydroxyl radical [53]. This radical is highly reactive with organic substances and can promote irreversible damage to enzymes and nucleic acids [54].

In the case of acetoin or diacetyl, both forms can coexist through oxireduction reactions, however, studies suggest that these compounds can interact with the arginine amino acid, compromising the structure of some proteins; despite the antagonistic action mechanism of diacetyl not being well established. In relation to diacetyl, another possible mechanism is that this compound can be able to link to DNA molecules, promoting its unfolding [55].

Up until twenty years ago, the exact mechanism of action for reuterin was undefined. This is because reuterin has an aldehyde compound in its molecular composition that is highly reactive and forms several other compounds in an aqueous solution. Thus, studies have characterized that this compound can induce oxidative stress in cells, through the modification of thiol groups in proteins or in small molecules [44, 56].

Regarding bacteriocins by definition are synthesized ribosomal antimicrobial peptides produced by bacteria and which have activity against another bacteria. Their activity can be between same species as narrow spectrum, or among other genera as broad spectrum. As a defense measure, bacteriocin-producing organisms are immune to their own bacteriocins [57, 58].

The bacteriocins studied can be classified and defined into classes: Class I (small modified peptides); Class II (unmodified peptides); and Class III (large peptides) > 10 kDa, that are based on their biosynthesis, mechanism, size, and type of molecule (Figure 2) [59, 60]. Also according to a significant increase in the characterization of new bacteriocins, there is a difficulty in performing the classification. Considering this, new class proposals will emerge [61]. Also, other types of bacteriocins, their mechanisms and classifications possible are well documented in a review presented by Lozo et al. [62]. According to the regulatory agency of the FDA (Food and Drug Administration) and the European Union, nisin is allowed for commercialization and others preparations containing pediocin PA-1 also can use in the food industry as food preservative [63, 64]. However, the application of nisin in food should be improved as it can interact with the food matrix (adsorption to salts, fat, and protein surfaces) and lose antimicrobial activity [65].

It is also important to note that although nisin is a biologically synthesized antimicrobial compound, perhaps it cannot be claimed as a clean label. This is because it is a food additive included and approved for use in foods (Code E234 for Europe) [66]. So, can generate confusion for a consumer, that is, when seeing the food additive claim in the list of ingredients, the consumer is induced to think that it is not something clean label.

Bacteriocins show several action mechanisms against microorganisms, and these are different from antibiotics. As already well documented in another review by Cotter et al. [67] and Lozo et al. [62], some bacteriocins, especially those that act on Gram-positive bacteria, work by targeting the cell wall. It is understood that some Class I bacteriocins link to lipid II of the cytoplasmatic membrane, preventing peptidoglycan synthesis. Despite blocking peptidoglycan synthesis, nisin can also insert into the cell membrane, forming pores. Some class II bacteriocins, such as lactococcin A, bind to the pore-forming receptor mannose phosphotransferase system (Man-PTS), and thus, eventually form pores. Bacteriocins, especially those that act on Gram-negative bacteria, have a particular mechanism of action which is based on the interference of protein synthesis, DNA and RNA; the action inhibits the production of DNA gyrase and RNA polymerase [67]. Indeed, the mechanism of action on Gram-negative bacteria is expected to be similar. The main point to be questioned is the contact of bacteriocin with the membrane due to the presence of an outer layer of lipopolysaccharide (LPS) in these bacteria. Several bacteriocins have been shown to be effective on Gram-negative, particularly when they are in combination with compounds that wash out the outer layer of bacteria. The combined use of bacteriocins with ethylenediaminetetraacetic acid (EDTA), for example, is one of the most common strategies for sensitizing Gram-negative bacteria. EDTA acts by promoting the release of the LPS layer and synergistically potentiates the antimicrobial activity of bacteriocins, as reviewed by Prudêncio et al. [68]. Recent studies report the use of bacteriocins with other compounds, as verified by Soltani et al. [61] that verified the synergistic effect of using Pediocin PA-1 bacteriocins combined with citric acid and/or lactic acid and which inhibited Gram-negative strains such as *Aeromonas hydrophila* and *Klebsiella pneumoniae*. In another study by Wang et al. [69] verified the synergistic effect of pediocin PA-1 with lactic acid which also inhibited the Gram-negative*A. hydrophila*.

In addition to inhibiting the growth of bacterial cells, some compounds produced by LAB are also related to the inhibition of fungal toxin production. According to Guimarães et al. [45], *L. plantarum* UM55 was able to inhibit the growth and aflatoxin production in five species of *Aspergillus.* According to the same authors, the absence of aflatoxin production is not associated to low fungal growth, but related to the production of phenylactic acid (PLA), hydroxyphenylactic acid (OH-PLA) and indolatic acid (ILA) by *L. plantarum* UM55. In addition to repressing fungal toxin production, compounds released by LAB also inhibit the growth of against some types of fungi. Zhao et al. [70] verified that compounds produced by *L. plantarum* were able to inhibit *Aspergillus Niger*, *Aspergillus oryzae*, *Trichoderma longibrachiatum*, *Aspergillus flavus* and *Fusarium graminearum.* In another study performed by Guimarães et al. [71], compounds produced by *L. plantarum* and *Lactobacillus buchneri* were able to prevent the growth of *Penicillium nordicum* as well ochratoxin production.

Although several studies demonstrate that LAB cultures have antagonistic activity on fungi and prevent toxin production in foods, the exact mechanism of action remains unclear for now [72, 73].

## 3. Steps for Identifying a Potential Bioprotective LAB

For the application LAB as a bioprotective culture in the food industry, a series of characteristics need to be assessed. These include; genetic stability; efficiency at low concentrations against a wide spectrum of pathogens and spoilage microorganisms in different food matrices; low nutritional requirements; survival in harsh environments, including food processing conditions; and not be pathogenic or toxic to humans [7, 74].

The development of new bioprotective LAB cultures with the potential to be commercialized and applied in the food industry requires several research steps which will be detailed in the following sections.

### 3.1. Isolation and Identification of LAB Strains

The LAB strains are distributed in a wide variety of ecosystems, including fresh and fermented foods, the gastrointestinal tract and mucosa of humans and animals, feces, water, pasture, leaf surfaces, rocks, the surface of equipment, and utensils used in food manufacture [29, 75–79]. Many studies suggest that LAB with antagonistic activity against pathogens and spoilage microorganisms can be isolated from fresh food, like milk, fruits, vegetables, fish, and even some meat products [13, 17, 29, 33, 62, 80].

Properly following the basic laboratory procedures for the isolation of a bacteria is essential to obtain success in the isolation and identification of a strain with desired characteristics and thus avoid problems such as loss of viability and changes in its antimicrobial activity. More information on these basic procedures can be found in a microbiological methods manual such as the one described by Da Silva et al. [81].

In general, all bacteria have specific biochemical and physiological growth requirements and, therefore, the formulated culture media must contain the required nutrients to support the microorganism growth. In regards to LAB, a considerable number of representative bacteria are fastidious, and require a rich and complex medium with different carbon sources [82–85]. LAB cultivation media generally has several sources of nitrogen (such as peptone, and yeast extract), minerals (such as Mn^2+^ and Mg^2+^), and buffering agents (such as sodium acetate) [85]. Monosaccharides are the main sources of energy and carbon required by bacteria, but other substrates can also perform these functions [86]. The Man Rogosa and Sharpe (MRS) medium is the most well-known and the oldest medium for LAB isolation, and was developed in 1960 for the selective cultivation of *Lactobacillus* species [87]. In 1975, Terzaghi and Sandine [88] developed the M17 medium for the cultivation of the *Streptococcus* bacteria genus. Since then, other specific media, such as the MSE was developed by Mayeux et al. [89], and MRS has had its composition revised and modified to support the growth of specific LAB [90, 91]. Likewise, Daniela et al. [91] enumerated *L. rhamnosus* and *L. acidophilus* on MRS supplemented with vancomycin and orclindamycin, while Vinderola and Reinheimer [90] enumerated *L. acidophilus* in MRS modified with trehalose.

Regarding LAB with bioprotective capacity, studies carried out using only standard media, such as MRS, M17 and MSE, with few modifications or supplementation were sufficient for the isolation of new strains [92, 93]. After isolation of new strains, verify the biochemical characteristics is the important point for select the specific bacteria. The biochemical tests are based on the biochemical characteristics of the bacteria, these include the production of enzymes like catalase and oxidase; capacity to ferment specific carbohydrates such as lactose, mannitol, maltose, fructose, glucose, xylose, and esculin; or the production of specific compounds such as diacetyl and acetoin. The biochemical tests aim to eliminate the colonies with biochemical characteristics that are different from LAB [94]. Despite biochemical tests being used in several research for reducing the number of colonies that need be genetically sequenced, these tests have been considered questionable, since LAB can assume different biochemical behaviors due to the constant genetic evolution of the strains in the environment [95]. In addition to genetic evolution, is know that most of the characteristics assumed by LAB are associated with the presence of plasmids in them and that they are important in carrying genes responsible for modifying and/or maintaining the characteristics of LAB in its ecological niche [96].

LAB screening is finished with the genome sequencing of all microorganisms that have gone through all the previous steps and, in this case, the bacterium is identified to the genus and species level [97]. Genome sequencing can be carried out using individual genes or the entire genome. In both cases, the data obtained by sequencing is compared with sequences deposited in the NCBI database (National Center for Biotechnological Information), allowing for the identification of the isolated microorganisms.

### 3.2. Characterization of Potential LAB Bioprotection Activity

Apart from being applied to LAB identification, genomic sequencing data can also be used to select potential bioprotective strains. Nowadays, at NCBI, more than 34,000 complete bacterial genomes are available, in addition to genes of interest [98]. Thus, through genome analysis, genes, operons, or gene groups of interest can be searched; these include those related to the synthesis of antimicrobial compounds; therefore, this ability can considerably favor the selection of a new bioprotective strain.

In a study carried out by Fusieger et al. [99], a strain of *Lactococcus lactis* subsp. *lactis* bv. diacetylactis had the complete gene cluster for nisin synthesis and export, similar to that of nisin Z. Urso et al. [100] demonstrated through sequencing and expression analyses the ability of a *L. sakei* strain to produce sakacin P. In another study carried out by Qi et al. [101], three complete gene clusters involved in the synthesis and secretion of homologous paracin bacteriocins and 7 clusters of new bacteriocins were identified in a strain of *L. paracasei*.

Although genomic research yields clues, genetic analysis does not rule out the need for in vitro or in situ (inside the food matrix) analysis. This is because a bacterium may have a gene related to the production of bacteriocins, organic acids, or any other antimicrobial compound but not express it under certain culture conditions.

Some studies demonstrate that LAB strains with bioprotection potential in food may have particular characteristics, such as the production of specific acids; these include succinic acid, vanillic acid, hydroxyisocaproic acid, and others with antifungal activity, such as phenylpropanoic acid, hydroxyphenylactic acid, propanoic acid, DL-pyroglutamic acid, 5-oxoproline, pidolic acid, and hydrocinnamic acid [102].

For characterization of the metabolite profile produced by LAB, chromatography is the most commonly used [103]. When compound identification is also linked to separation analysis, different types of techniques can be employed, including liquid chromatography (LC), gas chromatography (GC), capillary electrophoresis (CE), and supercritical gas chromatography (SFC) [104]. As an example, for the characterization of the organic acid profile, techniques such as GC-MS, which is gas chromatography coupled with mass spectrometry, can be explored. Bacteriocins can be characterized using HPLC–RP, which is a reversed-phase chromatography (RP-HPLC) technique, which separates components of a mixture by the difference in hydrophobicity (Table 2). Thus, these compounds can be detected, quantified, and fractionated.

For the chromatographic methods used in metabolite separation procedures, the most common systems in use are those based on the reverse phase (RP) and the hydrophilic interaction chromatograph (HILIC). RP-based methods are used to separate average and nonpolar metabolites, and the HILIC system is used for polar metabolites that cannot be retained in RP. In detection methods, systems coupled to mass spectroscopy (MS) are widely used; however, the efficiency of compound detection may depend on the matrix complexity and the analyte [102, 110].

Sharaf et al. [109] used the Headspace GC-MS technique for the characterization of several compounds produced by *Lactobacillus helveticus* and *L. plantarum.* Tian et al. [110] also used this same technique to identify volatile compounds, mainly diacetyl and acetoin produced by *L. plantarum.* Also, through an innovative and recent technique, biochromatography coupled with reversed-phasehigh-performance liquid chromatography (RP-HPLC), Pei et al. [107] managed to identify a new type of bacteriocin found in the cell suspension of *L. plantarum*.

### 3.3. Safety Aspects of Bioprotective Cultures

In order to have new bioprotective cultures on the market, it is important to highlight the risks and assess the safety aspects related to the strains. Despite some LAB showing beneficial effects on consumer health and having the GRAS status, certain strains can produce harmful substances, such as the biogenic amines, synthesize enzymes that degrade human's tissues, such as hemolysins, gelatinases, and cytolysins; and disseminate and/or transfer antibiotic resistance genes to pathogens [111].

Therefore, before a bacterium can be approved as a bioprotective culture, it is necessary to prove that the strain is safe for the host [10, 11]. As already mentioned, bacteria of the genus *Lactobacillus* have been historically used as bioprotective agents and probiotics, followed by *Streptococcus*, *Leuconostoc*, and *Pediococcus*, and are considered safe due to their history of use [112–115]. However, even in isolated cases involving patients with underlying medical conditions such as underweight neonates, adults, and babies in intensive care units and postoperative patients, these LAB genera have already been associated with systemic infections [112, 116–121].

Concerning these aspects, strain safety should be demonstrated through in vitro and in vivo testing. One of them includes the expression of virulence or antibiotic resistance genes, such as those in *Enterococcus* spp., which can carry virulence genes and express them when applied to the food matrix. One of the major concerns regarding microorganisms with antibiotic resistance genes is the horizontal transfer of these genes to other lactic acid cultures and/or other pathogenic bacteria [122–124]. Another is the research on virulence factors that include the production of biogenic amines, toxins, toxic metabolites, and enzymes such as hemolysins and gelatinase [112, 125]. Regarding the production of biogenic amines, these compounds have organic, heterocyclic, and aromatic bases. They are molecules that are generated primarily by the decarboxylation of their corresponding precursor amino acid [126, 127]. The amines have the potential to cause health risks to consumers by increasing blood pressure, causing food poisoning, and also reacting with nitrite to form carcinogenic nitrosamines [128]. When microorganisms have high proteolytic activity, the chances of biogenic amine formation increase due to the availability of free amino acids [129]. Furthermore, many lactic acid cultures are able to convert amino acids into biogenic amines, such as *Lactococcus* [130–132] and *Lactobacillus* [127, 132], through amino acid decarboxylation or transamination of aldehydes or ketones [133].

Also, is important the determination of hemolytic activity if the evaluated strain belongs to a species with known hemolytic potential [112, 125]. Hemolytic activity is an important factor in the selection of bioprotective and probiotic cultures, as it is associated with the ability of the strains to use the iron ions of red blood cells, which can trigger anemia and edema in the host [134]. The most common test for the determination of hemolytic activity in bacteria is based on the inoculation of these strains onto blood agar. The formation of halos around the colonies indicates a positive reaction to hemolytic activity, with clear halos indicating *β*-hemolysis, green halos to *α*-hemolysis, and the absence of halos determining *γ*-hemolysis [135, 136]. Finally, the production of gelatinase, which is considered a metalloendopeptidase, a proteolytic enzyme capable of hydrolyzing collagen, gelatin, insulin, casein, and other peptides [137, 138], the gelatinase substrates are identified in order to understand the function of these enzymes in the execution of their regulation, in which the main objective is to supply nutrients for the bacteria to cause different physiological and pathological responses in the host, such as vascular diseases, tumors, inflammation, infectious diseases, and degenerative diseases [139].

### 3.4. In Vitro and In Situ Tests

To validate if LAB strains are able to produce antagonistic substances, the adoption of in vitro tests is the simplest way to evaluate typical pathogenic and spoilage microorganisms [30, 32]. The in vitro tests are relatively easy, fast, and cheap to perform; however, no information is generated regarding the interaction between the antimicrobial substances and the food matrix [102].

After in vitro evaluation, the selected LAB strains should be analyzed by means of in situ inhibition bioassays, assessing if they have the capacity for biocontrol [26, 102, 140]. Regarding in situ inhibition, the assay can be experimentally designed to apply the potential bioprotective culture directly to the food or to incorporate it during its processing.

Aljasir et al. [141] evaluated the efficiency of individual and combined bioprotective cultures of *P. acidilactici*, *L. curvatus*, *L. plantarum*, and *Carnobacterium* spp. using a direct test on raw milk; they concluded that both the individual and combined culture tests had an antimicrobial effect on *L. monocytogenes*. Macieira et al. [41] applied *L. plantarum* to traditional Portuguese sausage and verified that the strain had an antagonistic effect on *L. monocytogenes*. Siroli et al. [29] demonstrated that *L. plantarum* was able to increase the shelf life of minimally processed sliced apples and lamb's lettuce for up to 9 days when used alone and up to 16 days when combined with other natural antimicrobials.

Besides incorporating the LAB strains directly into food, other in situ testing strategies involve the reproduction of the food matrix in a model system. This model system is normally created to optimize assays to reduce the time and price of analyses. Garnier et al. [142] tested the antifungal capacity of LAB cultures in a model system that mimics cheese (mini cheeses) in 24-well plates. The authors verified that there was antifungal activity in the tested LAB. However, as there were many tests performed as a way of optimizing analyses, it was noticed that this activity can vary according to the batches, manufacturing method, and care, among others. In the same context, Leyva Salas et al. [4] created models that imitated dairy cheese and yogurt to evaluate the antifungal activity of LAB combinations on some types of fungi. They found that two types of combinations were effective on fungi such as *Penicillium commune*, *Mucor racemosus*, and *Rhodotorula mucilaginosa*, both in in vitro and in situ tests [4].

Despite the substantial number of studies, few bioprotective cultures are available in the market due to restrictions such as the differences in effectiveness between in vitro and in situ assays, sensorial impacts on the food, safety, and maintenance of cell viability during commercialization [4, 41]. Therefore, tests at a pilot scale are essential to formulate an idea about a real industrial biocontrol scenario exercised by the culture and, thus, scale up its application for industrial production.

As an example of the discrepancies between in vitro and in situ tests, Delavenne et al. [143] detected 11 LAB strains with good antagonistic activity against fungi in in vitro tests. However, among the evaluated strains, only one showed high antagonistic activity when the in situ test was performed directly on the yogurt. Although some parameters can influence the behavior of a bioprotective culture in in vitro and in situ tests, in the case of the in vitro test, conditions such as time and incubation temperature, as well as the composition and/or modifications of the culture medium, can be preponderant factors for the strain that has good antagonistic effects [4]. Therefore, adjusting the time/temperature binomial and providing diverse cultivation conditions may or may not favor the bioprotective effect of the selected strain. Le Lay et al. [26] tested different compositions of culture media to verify the bioprotective activity of LAB. They identified great differences in antifungal activity between the evaluated culture media and also in the different sugar concentrations tested. They reported that the media, with a greater addition of concentrated sugars, also increased the production of organic acids that have a high antifungal effect.

Regarding in vivo tests, if a culture with bioprotective potential does not behave well as a bioprotective, one of the parameters that must be evaluated is whether this culture is in fact interacting with the food matrix, either for reasons of polarity or if something has been intentionally added, as some additives inactivate the bioprotective culture. In addition, another extremely important factor to consider is the concentration of the added bioprotective culture in the test, which can also strongly influence the bioprotective effect [26]. In these in vitro and in situ tests, any adverse factor must be eliminated for the culture to perform its bioprotection role well. And if in fact the culture presents a good bioprotective effect in both tests, the directive is that the next step be carried out, which is the application of tests in a pilot plant. In this way, the conditions for formulating the food product and applying the culture with bioprotective potential are closer to real conditions [4].

### 3.5. Pilot Tests

Once the bioprotective potential of a strain has been verified and proven in both in vitro and in situ tests, the last step is to carry out the tests on a pilot scale. This stage is carried out as one of the last phases of the strategy to identify a bioprotective culture. This step is performed last because it involves a greater amount of resources and aims to evaluate the potential with which the culture will meet the needs of the market. The pilot test tries to mimic the conditions of large-scale industrial production but is carried out on smaller-scale equipment (size and energy demand), also with the aim of reducing analysis costs and preventing the industrial processing plant from being paralyzed, even temporarily, to carry out the test. In these cases, the bioprotective culture can be added during product processing or inoculated during the final stages of processing, depending on the food product.

The main LAB cultures currently available on the market are for the production of cheese (Table 1), because these bacteria can produce acids that, when combined with the acids already naturally present in the product, will not significantly interfere with its sensorial characteristics [40]. Using cheese production as an example, in a pilot scale, following each step of the process is essential for maintaining the characteristics of the final product, as well as for the effectiveness of the protective culture. Some care is required when adding the protective culture, whether lyophilized or not; this includes considerations such as the temperature of the milk being close to the ideal action temperature of the culture (between 30 and 36°C), the pH being suitable, and the absence of antibiotics and contaminants that can influence the development of the culture. In general, the time for the adaptive response of the culture must also be considered. Therefore, before the addition of ingredients and the coagulation process, a minimum period of time (20 to 30 minutes) must be respected to adapt the culture to the environment. During production, some process parameters can also be evaluated (yield, fermentation time, curd, and pH) to assess the effect of the bioprotective culture on the process [9]. During ripening and storage time, some parameters can also be evaluated (degree of proteolysis, texture, ripening time if it is a ripening cheese, enumeration of fungi, and LAB).

The determination of shelf life is extremely important in this process, and the comparison between the product with the added and non-added culture must be carefully carried out to prove the effectiveness on a larger scale. Also, as proof of effectiveness, the sensory aspects need to be evaluated, as the bioprotective culture should not, or as little as possible, alter these attributes [9]. In this way, texture, color, aroma, flavor, and taste are prioritized in sensorial tests. Li et al. [40] evaluated the potential use of a strain of *L. casei* as a bioprotective culture in yogurt, and, in addition to the bioprotective effect, the authors also evaluated whether the culture influenced the attributes of color, texture, flavor, and taste. According to the sensorial test performed, no alteration in these attributes was significantly perceived. Cosentino et al. [144] evaluated attributes such as taste and aroma and found no significant differences between Caciotta cheese samples with or without bioprotective strains of lactobacilli. In another work, the authors verified that combinations of LAB cultures promoted significant differences in the perception of the acidity attribute of cheese and sour cream. However, the samples were accepted by consumers, indicating that although some differences were noticed, the final product was not completely modified and accepted [4]. Therefore, when adding a bioprotective culture, or combinations of them, checking the recommended dosage is essential so that an excess of metabolites is not produced to the point of modifying the sensorial characteristics of the product.

After these evaluations, if the protective characteristics of the culture are confirmed by the pilot tests and it is also verified that it does not influence the sensorial aspects of the food, the next step is to carry out the certification of the strains for commercialization, whose proceedings can vary from one country to another [145]. In the United States, the agency responsible for certifying microbial cultures for food application is the FDA, granting the well-known GRAS status [11]. The countries of America, as a whole, for culture certification, follow the recommendations governed by the FDA. In Europe, certification is made by the EFSA (European Food Safety Authority), which grants QPS (qualified presumption of safety) status [145].

A review carried out by Laulund et al. [146] presented some countries that have their own regulations for marketing within their own country, such as Japan, Thailand, China, and Malaysia. The first nation with national legislation that required safety approval for cultures applied to food was Denmark, and, in 2010, it no longer required approval for the marketing of cultures, but notification of a new strain (taxonomy) is required [145]. It is still a global challenge to certify ideal bioprotective cultures in the world market, considering that LAB cultures provide benefits to the food product, whether in fermentation or for some probiotic potential and an improvement of sensorial properties, and if safe, these can already be certified and marketed. However, current cultures with the bioprotective effect designation that can be certified for food application do not have the necessary efficacy. This certification step is the last of the development of bioprotective cultures in the food preservation process, and, therefore, after certification, the process of sales and marketing begins.

## 4. Conclusion

Independent of the studies and complete characterization of the compound profiles, the protective effect of biopreserving cultures can suffer variations in different and complex food matrices and with different species and their combinations. It is still a challenge to develop bioprotective cultures with significant effects due to the complexity of interactions and food matrices. In addition, granting GRAS or QPS status requires complete studies that are often not in accordance with what the law requires. Thus, more research is needed to understand the performance of metabolites in bioprotection in the complex environment of food matrices. In addition, cultures can be combined with other methods of preservation and synergy as a way to ensure their effectiveness.

The current challenge in the development of effective protective cultures lies in identifying the compounds produced in the different food matrices and their action/efficacy on different types of target microorganisms. Many of the compounds were not detected by the techniques employed; however, advancement in the sensibility and precision of the analytical tools can be the key factor in isolating and identifying potential compounds with antimicrobial activity.

## Figures and Tables

**Table 1:** Major cultures used as bioprotective in food products.

Research studies			
Application product	Microorganism	Target microorganisms	References
Marine products			
Cold-smoked salmon	*Latilactobacillus sakei * ^1^	*Listeria monocytogenes*	[12]
	*Latilactobacillus curvatus * ^2^		[12]
	*Carnobacterium maltaromaticum*		[12]
Fish pâté	*Lactococcus lactis* subsp. *Lactis*	*Vibrio sp.*	[13]
Gilt head sea bream	*L.sakei * ^1^	*Listeria monocytogenes*	[14]
Tuna burgers	*Lacticaseibacillus paracasei * ^3^	*Pseudomonas spp.*	[15]

Meat			
Cooked ham	*Lactiplantibacillus plantarum * ^4^	*Bacillus cereus*	[16]
Cooked ham	*Pediococcus acidilactici*	*Clostridium sp. like botulinum*	[16]
Fresh sausage	*L. curvatus * ^*2*^	*Listeria monocytogenes*	[17]
Chicken breast	*Enterococcus lactis*	*Listeria monocytogenes*	[18]
Meatballs	*Pediococcus acidilactici*	*Escherichia coli *0157:H7	[19]
Fermented sausage	*L. curvatus * ^2^	*Listeria monocytogenes*	[20]
Speck	*Debaryomyces hansenii*	*Aspergillus ochraceus/P. nordicum*	[21]
Speck	*Saccharomycopsis fibuligera*		[21]
Suckling-lambmeat	*Leuconostoc pseudomesenteroides*	*Listeria monocytogenes*	[22]

Bakery products			
Pan whole-wheat	*Limosilactobacillus reuteri * ^5^	*Aspergillus niger*	[23]
Panettones	*Limosilactobacillus fermentum * ^6^	*Molds and yeasts*	[24]
Sourdough bread	*L. paracasei * ^3^	*Bacillus spp.*	[25]
Pound cake	*L. reuteri * ^5^	*Cladosporium sphaerospermum*	[26]
Pound cake	*Levilactobacillus spicheri * ^7^	*Cladosporium sphaerospermum*	[26]
Milk bread rolls	*Lactobacillus citreum*	*Aspergillus niger*	[26]

Fruits and vegetables			
Table olive	*L. plantarum * ^4^	*Listeria monocytogenes*	[27]
Cabbage	*L. plantarum * ^4^	*Listeria monocytogenes*	[28]
Apples	*L. plantarum * ^4^	*Escherichia coli/Listeria monocytogenes*	[29]
Apples	*Leuconostoc mesenteroides*	*Listeria monocytogenes*	[30]
Pickles	*L. plantarum * ^4^	*Candida albicans*	[31]
Orange	*Weissella paramesenteroides*	*Penicillium digitatum*	[32]
Orange	*Liquorilactobacillus sucicola * ^8^	*Penicillium digitatum*	[32]
Grape bunch	*Pediococcus pentosaceus*	*Aspergillus niger/Aspergillus carbonarius*	[30]
Lettuce	*Leuconostoc mesenteroides*	*Listeria monocytogenes*	[33]

Dairy products			
Cheese feta	*Lactobacillus acidophilus*	*Aspergillus niger*	[34]
Sour cream and cheese	*L.plantarum * ^4^ * /Lactobacillus harbidenses*	*Penicillium commune/Mucor racemosus*	[4]
Sour cream	*L. plantarum * ^4^	Penicillium commune	[35]
Fermented milk	*L. harbidenses*	Yarrowia lipolytica	[36]
Gorgonzola cheese	*Lactococcus lactis* subsp. *Lactis*	*Listeria monocytogenes*	[37]
Fresh cheese	*Lactococcus lactis* subsp. *Lactis*	Bacillus cereus	[38]
Kasseri cheese	*Streptococcus macedonicus*	Clostridium tyrobutyricum	[39]

Commercial cultures available on the market			
Product/manufacturer	Application	Target microorganisms	Composition

FreshQ®1/CHR Hansen	Fermented dairy products	Yests and molds	*Lacticaseibacillus rhamnosus^9^* and *L. paracasei^3^*
FreshQ®2/CHR Hansen	Fermented dairy products	Yests and molds	*L. rhamnosus^9^* and *L. paracasei^3^*
FreshQ®4/CHR Hansen	Fermented dairy products	Yests and molds	*L. rhamnosus^9^* and *L. paracasei^3^*
SafePro® B-LC-007/CHR Hansen	Fermented sausage	*Listeria monocytogenes*	*Debaryomyces hansenii, L. sakei^1^*, *Pediococcus acidilactici, Pedio Listeria monocytogenes coccus pentosaceus, Staphylococcus carnosus, Staphylococcus xylosus*
Viniflora®CH16/CHR Hansen	Wine	Yests and molds	*Oenococcus oeni*
Concerto^TM^/CHR Hansen	Wine	Yests and molds	*Lachancea thermotolerans*
Delvo®Guard 201/DSM	Soft cheese	Yests and molds	*L. rhamnosus^9^* and *L. sakei^1^*
Delvo®Guard 301/DSM	Soft cheese	Yests and molds	*L. rhamnosus^9^*
Lyofast LPR A/SACCO	Cheese hard and semi-hard	Yests and molds	*L. rhamnosus^9^* and *L. plantarum^4^*
Bioprox RP80/PROXIS	Dairy products	Yests and molds	*L. rhamnosus^9^* and *L. plantarum^4^*
Lyopro ® Tect/Codex-ing	Fermented milk and cheese	Yests and molds	*L. rhamnosus^9^* and *Propionibacterium shermanii*
HOLDBACK® Listeria dairy/Danisco	Soft and smear cheese, dry and semi-dry cured meats, cooked and fresh ground meats	*Listeria monocytogenes*	*L. plantarum^4^*
HOLDBAC® YM-XPM/Danisco	All cheese types	Yests and molds	*L. paracasei^3^* and *L. plantarum^4^*
M-CULTURE® Safe 1100/Meatcracks	Raw sausages	*Listeria monocytogenes*	*Staphylococcus carnosus*, *L. curvatus^2^* and *Staphylococcus xylosus*
Protek/BIOCHEM	Fresh and soft cheese	Yests and molds	*L. rhamnosus^9^*

^1^ Previous *Lactobacillus sakei*. ^2^ Previous *Lactobacillus curvatus*. ^3^ Previous *Lactobacillus paracasei*. ^4^ Previous *Lactobacillus plantarum*. ^5^ Previous *Lactobacillus reuteri*. ^6^ Previous *Lactobacillus fermentum*^7^ Previous *Lactobacillus spicheri*. ^8^ Previous *Lactobacillus sucicola*. ^9^ Previous *Lactobacillus rhamnosus*.

**Table 2:** Application of techniques for identification of compounds from bioprotective cultures.

Technique employed	Some detected metabolite	Reference
HPLC	Lactic, citric, acetic, succinic	[102, 105]
	PLA, OH-PLA acids	[106]

HPLC-RP	Benzoic	[103]
	Bacteriocin	[107]

ESI-MS/MS	Short polycyclic lactates	[103]
GC-MS	6-octadecenoic acid methyl ester, hexadecanoic acid methyl ester, phenol, 2-4 bis (1,1 dimethylethyl)	[108]
GC-FID	Stearic, palmitic, oleic, mystiric caproic, caprylic acids	[102]
HS-GC-MS	7-methyl-Z-tetradecen-1-ol acetate, 9-Hexadecenoic acid, 9-Octadecenamide	[109]
